# Editorial: Combating Bacterial Infections Through Biomimetic or Bioinspired Materials Design and Enabling Technologies

**DOI:** 10.3389/fbioe.2021.818643

**Published:** 2021-12-16

**Authors:** Gianluca Ciardelli, Aldo R. Boccaccini, Ipsita Roy, Antonia Nostro, Jochen Salber

**Affiliations:** ^1^ Department of Mechanical and Aerospace Engineering, Politecnico di Torino, Turin, Italy; ^2^ Institute of Biomaterials, University of Erlangen Nuremberg, Erlangen, Germany; ^3^ Department of Materials Science and Engineering, The University of Sheffield, Sheffield, United Kingdom; ^4^ Department of Chemical, Biological, Pharmaceutical and Environmental Sciences, University of Messina, Messina, Italy; ^5^ Salber Laboratory, Centre for Clinical Research, Department of Experimental Surgery, Ruhr-Universität Bochum, Bochum, Germany; ^6^ Department of Surgery, Universitätsklinikum Knappschaftskrankenhaus Bochum GmbH, Bochum, Germany

**Keywords:** biomimetic polymers, *in vitro* models, surface functionalisation, bioactive ceramic, antibacterial activity, bioinspired biomaterials, drug-free, antibacterial composites

An essential cornerstone of modern medicine for the implementation of curative surgical or interventional therapies and for the treatment of microbial, bacterial and fungal infections was the discovery and development of antibacterial (antibiotics) and fungicidal (antifungals) agents that could be applied systemically either orally or parenterally (intravenously). In addition, improving hygiene in hospitals before, during and after invasive procedures (working aseptically) has significantly reduced the number of postoperative infections. With the introduction of antibiotics and antifungals, it was discovered almost simultaneously that these substances lead to ineffectiveness in various microbes or strains of these microbes (Ventola, 2015). In other words, certain subpopulations of microbes developed resistance to these newly introduced antimicrobial agents as a result of a natural evolutionary process with increasing drug use and spread. In this way, a kind of race developed between the development of new antimicrobials, especially antibiotics and the development of resistance of microbes, especially bacteria, to these agents. In the course of the further development of medicine with new therapeutic possibilities such as the steadily increasing implantation of foreign materials (such as catheters, stents, joint endoprosthesis and many more) as well as technical possibilities to perform increasingly complex operations (e.g., limb reconstructions, transplantations, etc.) or life-sustaining, intensive medical therapies on a daily basis, the use and consumption of antibiotics and antimycotics also increased (Charani et al., 2017).

However, not only in human medicine, but also in other areas, increasingly larger quantities of antimicrobial agents and antiseptic disinfectants were used and in many cases misused. This attitude has led to a steadily growing number of resistant bacterial and fungal species. New development and approval of effective antimicrobial agents has not kept pace with this drastic development (Hutchings et al., 2019). With the increasing use of foreign materials (biomaterials, wound dressings, implants, endoprosthesis) in patients and the growing number of patients with chronic wounds, another phenomenon has been discovered, the biofilm formation of bacteria and fungi on foreign body surfaces or on necrotic or immune-compromised host tissues. As part of the complex biofilm formation process, microbes create a survival habitat in which they can evade the influence of the host’s own immune system, largely protect themselves from the effects of applied antibiotics and antifungals, and even develop resistance to these agents (Idrees et al., 2018).

All this led to the consideration not only of the development and approval of new antibiotics and antifungals as a target-oriented strategy, but also to look for other alternative and complementary strategies. Just as the first so-called classical antibiotic penicillin was produced by a fungal culture (i.e., by nature itself), there is an almost inexhaustible repertoire of natural substances such as antimicrobial peptides (AMPs), biofilm-degrading enzymes, inorganic ions and principles such as anti-adhesive surface modifications or morphologies for the prevention of bacterial adhesion and thus biofilm formation or surface-active destruction of microbes (Amin Yavari et al., 2020). All this led to the fact that today we are in the so-called antimicrobial resistance (AMR) crisis. This is where our Research Topic *Combating Bacterial Infections Through Biomimetic or Bioinspired Materials Design and Enabling Technologies* comes in, to harness the natural potential of antimicrobial strategies for modern medicine. As is often the case when exploring new ways to solve an existing problem, such as foreign body-associated infections, biofilm formation and resistance to classical antibiotics, new challenges are brought to light. This includes the proof of efficacy of new alternative strategies and filling gaps in understanding of the interaction of complex multimicrobial aggressors with the host. In addition, modelling of these complex interactions and preclinical testing of these new compounds and/or strategies *in vitro* is required. This will ensure that the biofilms can be prevented or removed, and the microbes killed without at the same time harming the host cells and thus the host itself. In this research topic, a review is devoted to illustrate these challenges (Oriano et al.). In addition, further 13 original papers highlight various strategies for combating biofilms and clinically relevant bacterial strains, some of which are highly resistant, in an alternative-complementary manner.

Reactive oxygen species (ROS) in general and hydrogen peroxide in particular are generally considered to be potential antimicrobial agents. Clinically, dilute aqueous hydrogen peroxide solutions have been widely used in the cleansing of heavily contaminated soft tissue wounds or in the sanitation of bony implant regions in the case of periprosthetic joint infections (PJI). Here, the detrimental effect on the local host cells, whose physiological functioning is extremely important for healing, was disregarded for a long time. This does not mean that hydrogen peroxide or other ROS are unsuitable, because even certain defense cells (e.g., phagocytes) in the human body make use of them, but in a clever, intelligent way. In the article by Vasquez et al. it is shown how the antimicrobial effect of honey can be cleverly imitated by material engineering to release defined hydrogen peroxide concentrations locally (e.g., in wounds) to kill bacteria without damaging the host cells. In another original work, Mancuso et al. combined Manuka honey (MH, polyanionic), which is recognized as having antimicrobial activity, with poly-(allylamine hydrochloride) (polycationic) as a LbL coating using electrospun PCL meshes as a substrate to generate a biocompatible, antimicrobial, and multilayer nanocoating on the PCL mesh. Among other things, the authors were able to show that the LbL technique can be used as an effective means for the production of complementary antimicrobial medical devices.

In connection with open bone fractures, penetrating contaminants and thus bacteria, surgeons are faced with the major problem of eliminating bacterial entry, preventing bacterial adhesion and thus biofilm formation on the implants and especially at the implant-bone interface, replacing lost bone substance and at the same time supporting fracture healing and bone regeneration, in addition to acute bone fracture treatment with temporary force-bearing implants (intramedullary nails, plate-screw systems, etc.). This is where the work of Marcello et al. comes in and reported for the first time the synthesis of a novel dual-substituted hydroxyapatite (HA) containing selenium (Se) and strontium (Sr) and its combination with two types of polyhydroxyalkanoates (PHAs) differing in molecular chain length (short-chain, scl; medium-chain, mcl). From this, the authors prepared novel antibacterial composite materials for bone regeneration without using antibiotics. Modification of the PHA matrix with the antibacterial Se- and bone regenerative Sr-containing HA resulted in an increase in the mechanical strength of both scl- and mcl-PHA films while decreasing the deformability of the materials. These newly developed composite materials possess high antibacterial activity against *Staphylococcus aureus* 6538P and *Escherichia coli* 8739. In an interesting paper by Thijssen et al., the authors approached the problem of chronic osteomyelitis from a translational, clinically practical perspective. They optimized the handling properties of a clinically used bioglass-based antimicrobial bone substitute S_53_P_4_ (Bonalive^®^) to facilitate its use by orthopedic surgeons. The authors tested these new injectable putty formulations on five different clinically relevant germs and investigated the influence of the binder components poly(ethylene)glycol (PEG) and glycerol on the antibacterial properties of the bioactive glass S_53_P_4_. The authors critically discussed the results previously presented in the literature as well as their own.

Monovalent silver (Ag^+^) ion releasing systems continue to be a clinically used strategy to prevent bacterial adhesion to implant surfaces or to combat bacteria in the wound area. Due to their concentration-dependent toxicity to mammalian cells, nanoparticulate silver release systems in particular represent an interesting solution. Aabed and Mohammed showed in their article that biogenic AgNPs capped and reduced by biomolecules such as carbohydrates and proteins result in safe, inexpensive and stable Ag^+^-releasing nanoparticles that could be useful in antimicrobial drug formulations. Nevertheless, it is important to critically investigate the use of alternative, complementary antimicrobial strategies such as the application of inorganic ion-releasing nanoparticles (Ag^+^ in this case) with respect to their mechanism of action. It is important not only to understand their microbe-killing effect, but also to shed light on their eukaryotic toxicity and immunomodulatory properties. In another contribution to this topic Varela et al. addressed this issue and investigated various clinically used wound care materials that can release silver ions with respect to cytocompatibility and immunomodulation *in vitro*. In particular, subchronic and chronic wounds with often severe microbial colonization and complex inflammatory status, which is individually dependent on the overall health of the affected patient, represent a major challenge and socioeconomic burden. This is a significant market for the medical device industry and there are now many high-tech products on the market. Therefore, it is not surprising that basic experimental research is viewed critically by the industry. This critical bench-to-market-to-clinic-and-back discussion is tremendously important to better understand and optimize the efficacy of current antimicrobial wound dressings and their investigations.

The enormous importance of the topic “chronic wounds and antimicrobial wound dressings” is also illustrated by the fact that three additional research articles are dedicated to this topic within the scope of this special edition. The work by Ward et al. describes for the first time a novel mcl-PHA/graphene composite wound patch that can detect the presence of infection with *Pseudomonas aeruginosa* PA_14_, an important wound pathogen, in real time. In addition, a closed-loop control algorithm was outlined that will be further developed to create a system that can dynamically control the concentration of antimicrobial agents (such as silver ions, but also others) in a wound bed to actively kill *P. aeruginosa* in response to infection. Orlando et al. have presented for the first time a promising strategy for using bacterial cellulose as a wound dressing with tailored properties. Its intrinsic hydrogel-like nature can provide a high level of moisture for dry wounds and great biocompatibility, while the eco-friendly functionalization performed can ensure inhibitory activity against various bacterial species. In addition, this versatile method can also be applied to other polysaccharides to provide them with intrinsic antibacterial properties while improving their wound healing properties.

Essential oils represent another group of substances that may have anti-microbial as well as anti-inflammatory properties. Here, too, it is crucial how such substances are formulated, applied and released. Unalan et al. combined peppermint essential oil (PEP) with poly (ɛ-caprolactone) (PCL) in electrospun fiber mats for use as cytocompatible and antimicrobial wound dressings. Leyva Del Rio et al. used tt-farnesol, a natural sesquiterpene alcohol found in propolis, as a potential antimicrobial and anti-biofilm agent in dental adhesive systems. Formulation of this agent was shown to suppress both the production of extracellular insoluble polysaccharides (EIPs) and the bacterial viability of *Streptococcus mutans* biofilm. However, the authors also critically discussed that the addition of tt-farnesol to the complex composition of the dental adhesive system altered its physical and binding properties.

Two other research papers investigated the usability of AMPs. While Najmi et al. investigated the use of the antimicrobially active peptides Nisin and LL-37 to prevent septic articular cartilage degeneration, Piarali et al. dealt with the development of electrospun PHA meshes modified in a single step with a combination of the AMP Amhelin and the biofilm degrading enzyme Dispersin B. Both works were able to demonstrate antimicrobial (and biofilm-disruptive) efficiency while maintaining biocompatibility considering application and environmental conditions.

Amphiphilic cationic macromolecules (ACMs) represent another promising way to attack bacteria and fungi, including highly resistant strains, and their polymicrobial biofilms. For example, Mukherjee et al. demonstrated that their polyetheleneimine-based ACMs are highly active against MRSA and *Candida albicans* without damaging mammalian cells in the required therapeutic window.

In all cases, Nature itself is the inspiration. More specifically, *Combating Bacterial Infections Through Biomimetic or Bioinspired Materials Design and Enabling Technologies* invites the reader to learn about breakthrough contributions from leading scientists in the areas of i) Biomimetic polymers and hybrids with antibacterial activity, ii) Bioactive ceramics and glasses that prevent bacterial growth, iii) Design of unfavorable surface topographies and chemical functionalisation for bacterial colonization, and iv) 2D and 3D *in vitro* testing of antibacterial activity and eukaryotic biocompatibility.
